# Determinants of delay in diagnosis and end stage at presentation among breast cancer patients in Iran: a multi-center study

**DOI:** 10.1038/s41598-020-78517-6

**Published:** 2020-12-08

**Authors:** Elahe Foroozani, Reza Ghiasvand, Mohammad Mohammadianpanah, Sima Afrashteh, Dariush Bastam, Fatemeh Kashefi, Saba Shakarami, Mostafa Dianatinasab

**Affiliations:** 1 School of Physical and Occupational Therapy, McGill University, Montreal, Canada; 2 Oslo Centre for Biostatistics and Epidemiology, Oslo University Hospital, Oslo, Norway; 3 Department of Research, Cancer Registry of Norway, Oslo, Norway; 4 Colorectal Research Center, Faghihi Hospital, Shiraz University of Medical Sciences, Shiraz, Islamic Republic of Iran; 5 Department of Epidemiology, Faculty of Public Health, Shiraz University of Medical Sciences, Shiraz, Islamic Republic of Iran; 6 Deputy of Health, Mazandaran University of Medical Sciences, Sari, Islamic Republic of Iran; 7 Department of Epidemiology, Faculty of Public Health, Ilam University of Medical Sciences, Ilam, Islamic Republic of Iran; 8 Medical School, Yasuj University of Medical Sciences, Yasuj, Islamic Republic of Iran

**Keywords:** Cancer, Breast cancer, Cancer epidemiology, Cancer screening

## Abstract

One of the reasons for high mortality of breast cancer (BC) is long delay in seeking medical care and end stage at presentation. This study was designed to measure the association between a wide range of socio-demographic and clinical factors with diagnostic delay in BC and stage at presentation among Iranian patients. From June 2017 to December 2019, 725 patients with newly diagnosed BC in Shiraz and Kermanshah were selected and information on BC diagnosis delay was obtained from the patient’s medical record. Data on socio-economic status was obtained via a structured interview. Our findings suggest that 45.8% of the patients were diagnosed at a late stage (stage 3 or higher). A total of 244 (34%) patients had more than 3 months delay in diagnosis. We found a significant association between stage at diagnosis and place of residence (adjusted odds ratio (aOR rural vs. urban = 1.69, 95% CI 1.49–1.97), marital status (aOR 1.61, 95% CI 1.42–1.88), family history of BC (aOR 1.46, 95% CI 1.01–2.13), and history of benign breast disease (BBD) (aOR 1.94, 95% CI 1.39–2.72) or unaware of breast self-examination (BSE) (aOR 1.42, 95% CI 1.42–1.85), delay time (aOR 3.25, 95% CI 1.04–5.21), and left breast tumor (aOR right vs. left 2.64, 95% CI 1.88–3.71) and smoking (aOR no vs. yes 1.59, 95% CI 1.36–1.97). Also, delay in diagnosis was associated with age, family income, health insurance, place of residence, marital status, menopausal status, history of BBD, awareness of breast self-examination, type of first symptoms, tumor histology type, BMI and comorbidity (p < 0.05 for all). Factors including history of BBD, awareness of BSE, and suffering from chronic diseases were factors associated with both delay in diagnosis and end stage of disease. These mainly modifiable factors are associated with the progression of the disease.

## Introduction

Although the incidence of breast cancer (BC) is higher in high-income countries, the majority of BC-related deaths occur in low- and middle-income countries^1^. It is reported that while more than 70% of breast cancer patients in high-income countries are diagnosed in the stage 1 and 2, corresponding proportion in low- and middle-income countries is about 20–50%^2^. Delayed diagnosis and treatment of BC are associated with a poorer survival^3,4^, and amongst the most important reasons for the significant differences in the mortality rate of BC in different countries^5^.

Although BC is a major health problem in Western Europe, North America and Australia^6^, it's incidence has significantly increased in many Asian countries, including Iran^7^. In fact, the estimated annual incidence of BC in Iran is approximately 20 new cases per 100,000 women^8^, of whom 70% are being diagnosed in end stage and die within a short period of time^9^.

Delay in diagnosis and treatment of BC is divided into four categories: patient delay (the interval between symptom onset and first consultation with physician), health or medical care provider delay (from first consultation to initiation of the treatment), delay in service provider (from first consultation to pathological confirmation of the diagnosis of BC), and eventually treatment delay (from pathological confirmation of the diagnosis to initiation of the treatment)^10^. Clinically, delayed diagnosis of BC has been defined as of three months or more delay in diagnosis, which is associated with poor prognosis^11^. Several factors are found to be associated with delay in diagnosis of BC, including socioeconomic status, age, health insurance, menopause, tumor type, breast self-examination (BSE), and marital status^12–18^. However, most studies on diagnostic delay of BC have been conducted in developed countries, which are different from developing countries in many aspects including cultural and health behaviors and life style^19,20^. Delay in diagnosis may not only decrease the survival of the patient, but may also increases the medical costs, more invasive treatments and reduced quality of life^21^. Thus, identification of factors associated with diagnosis delay of BC is crucial in providing more effective treatment and care^22^.

Due to limited evidence on determinants of delay in BC diagnosis in women from Asia, especially in Western Asia and Iran, this study was aimed to investigate and identify factors associated with delayed BC diagnosis and late stage presentation of women with BC.

## Results

### General characteristics

Table 1 represents the characteristics of the 725 BC participants according to delay in diagnosis. The patients’ mean age at diagnosis was 43.7 years (standard deviation 8.2). Family history of BC was reported among 189 (25.2%) of the participants and about 40% of women had a history of benign breast disease. The most frequent histological subtype was ductal carcinoma 522 (70%) followed by lobular carcinoma 136 (18%).

**Table 1:** Characteristics of the study participants according to the delay in diagnosis (n = 750) among Iranian women, 2017–2019.

Variables	Delay in diagnosis			Total n(%)
	< 30 days n(%)	30–90 days n(%)	> 90 days n(%)	
**Age (years)**				
< 40	65 (26.75)	77 (29.28)	67 (27.46)	209 (27.87)
40–50	84 (34.57)	86 (32.70)	102 (41.80)	272 (36.26)
50–60	73 (30.04)	80 (30.42)	57 (23.36)	210 (28.00)
> 60	21 (8.64)	20 (7.60)	18 (7.38)	59 (7.87)
**Education**				
Primary and lower	94 (38.68)	110 (41.83)	107 (43.85)	311 (41.47)
Middle school	64 (26.34)	91 (34.60)	79 (32.38)	234 (31.20)
High school	46 (18.93)	33 (12.55)	40 (16.39)	119 (15.87)
College	39 (16.05)	29 (11.03)	18 (7.38)	86 (11.46)
**Occupation**				
Housewife	178 (73.25)	194 (73.76)	186 (76.23)	558 (74.40)
Employed	65 (26.75)	69 (26.24)	58 (23.77)	192 (25.60)
**Family income**				
Poor	39 (16.05)	73 (27.76)	107 (43.85)	219 (29.20)
Moderate	97 (39.92)	98 (37.26)	75 (30.74)	270 (36.00)
High	107 (44.03)	92 (34.98)	62 (25.41)	261 (34.80)
**Health insurance**				
No	33 (13.58)	59 (22.43)	88 (36.07)	180 (24.00)
Yes	210 (86.42)	204 (77.57)	156 (63.93)	570 (76.00)
**Place of residence**				
Rural	80 (32.92)	109 (41.44)	93 (38.11)	282 (37.60)
Urban	163 (67.08)	154 (58.56)	151 (61.89)	468 (62.40)
**Marriage status**				
Single (never married)	47 (19.34)	104 (39.54)	75 (30.74)	226 (30.13)
Ever married	196 (80.66)	159 (60.46)	169 (69.26)	524 (69.87)
**Marriage age**				
< 20	80 (32.92)	77 (29.28)	62 (25.41)	219 (29.20)
20–25	52 (21.40)	37 (14.07)	22 (9.02)	111 (14.80)
25–30	31 (12.76)	46 (17.49)	36 (14.75)	113 (15.06)
> 30	15 (6.17)	27 (10.27)	39 (15.98)	81 (10.80)
Not married	65 (26.75)	76 (28.90)	85 (34.84)	226 (30.13)
**Age at first childbirth**				
< 20	107 (44.03)	92 (34.98)	94 (38.52)	293 (39.06)
20–30	87 (35.80)	89 (33.84)	78 (31.97)	254 (33.86)
> 30	25 (10.29)	49 (18.63)	40 (16.39)	114 (15.20)
Single or no child	24 (9.88)	33 (12.55)	32 (13.11)	89 (11.86)
**Menopause status**				
Postmenopausal	60 (24.69)	105 (39.92)	115 (47.13)	280 (37.33)
Premenopausal	183 (75.31)	158 (60.08)	122 (52.87)	463 (61.73)
**Family history of BC**				
No	169 (69.55)	204 (77.57)	188 (77.05)	561 (74.80)
Yes	74 (30.45)	59 (22.43)	56 (22.95)	189 (25.20)
**History of breast problem**				
No	166 (68.31)	159 (59.32)	130 (53.28)	455 (64.53)
Yes	77 (31.69)	107 (40.68)	114 (46.72)	298 (39.73)
**Aware of self-examination**				
No	86 (35.39)	189 (71.86)	201 (82.38)	476 (63.46)
Yes	157 (64.61)	74 (28.14)	43 (17.62)	274 (36.53)
**Type of first symptom**				
Lump	128 (52.67)	97 (36.88)	69 (28.28)	294 (39.20)
Discharge and pain	84 (34.57)	126 (47.91)	125 (51.23)	335 (44.66)
Other (by screening)	31 (12.76)	40 (15.21)	50 (20.49)	121 (16.13)
**Type of tumor**				
Ductal	192 (79.01)	186 (70.72)	144 (59.02)	522 (69.60)
Lobular	29 (11.93)	50 (19.01)	57 (23.36)	136 (18.33)
Other	22 (9.05)	27 (10.27)	43 (17.62)	92 (12.26)
**Stage**				
Early stage	161 (66.26)	140 (53.23)	105 (43.03)	406 (54.13)
Late stage	82 (33.74)	123 (46.77)	139 (56.97)	344 (45.86)
Location				
Right	145 (59.67)	134 (50.95)	109 (44.67)	388 (51.73)
Left	98 (40.33)	129 (49.05)	135 (55.33)	362 (48.26)
**X_ray history**				
No	213 (87.65)	223 (84.79)	203 (83.20)	639 (85.20)
Yes	30 (12.35)	40 (15.21)	41 (16.80)	111 (14.80)
**Daily exercise (min)**				
< 10	79 (32.51)	146 (55.51)	173 (70.90)	398 (53.06)
10–20	107 (44.03)	98 (37.26)	47 (19.26)	252 (33.60)
> 20	57 (23.46)	19 (7.22)	24 (9.84)	100 (13.33)
**BMI (kg/m**^SPS:refid::bib22^**)**				
Underweight (< 18.50)	42 (17.28)	32 (12.17)	44 (18.03)	118 (15.73)
Normal (18.50–24.99)	88 (36.21)	50 (19.01)	49 (20.08)	187 (24.93)
Overweight (24.99–29.99)	64 (26.34)	122 (46.39)	98 (40.16)	284 (37.86)
Obese (≥ 30.00)	49 (20.16)	59 (22.43)	53 (21.72)	161 (21.46)
**Chronic disease**				
No	181 (74.49)	119 (45.25)	151 (61.89)	451 (60.13)
Yes	62 (25.51)	144 (54.75)	93 (38.11)	299 (39.86)
**Smoking**				
No	214 (88.07)	227 (86.31)	205 (84.02)	646 (86.13)
Yes	29 (11.93)	36 (13.69)	39 (15.98)	104 (13.86)

^+^The status of variables at the diagnosis of cancer.

### Associations between study variables and end-stage diagnosis of BC

Women residing in rural areas were more likely to be diagnosed with end-stage of BC [adjusted odds ratio (aOR) 1.69, 95% confidence interval (CI) 1.49–1.97]. Also, being single (aOR 1.61, 95% CI 1.42–1.88), menopaused (aOR 1.29, 95% CI 1.20–1.41), a family history of BC (aOR 1.46, 95% CI 1.01–2.13), history of benign breast disease (aOR 1.94, 95% CI 1.39–2.72), and knowledge of BSE (aOR 0.42, 95% CI 0.42–0.85) were associated with end-stage diagnosis of BC (Table 2).

**Table 2:** Unadjusted and adjusted association between the study variables and end-stage diagnosis of breast cancer among Iranian women. 2017–2019.

Variables	Unadjusted^a^			Adjusted^b^		
	OR	95% CI	P value	OR	95% CI	P value
**Place of residence**						
Rural	Ref	–	0.26	Ref	–	0.03
Urban	0.84	0.61, 1.15		0.69	0.49, 0.97	
**Marriage status**						
Single (never married)	Ref	–	0.632	Ref	–	0.009
Ever married	0.92	0.66, 1.28		0.61	0.42 , 0.88	
**Menopause status**^c^						
Postmenopausal	Ref	–	> 0.001	Ref	–	< 0.001
Premenopausal	0.48	0.35–0.66		0.29	0.20 , 0.41	
**Family history of BC**						
No	Ref	–	0.263	Ref	–	0.04
Yes^d^	0.81	0.56–1.16		1.46	1.01 , 2.13	
**History of breast problem**						
No	Ref	–	0.696	Ref	–	< 0.001
Yes	1.06	0.77, 1.43		1.94	1.39 , 2.72	
**Aware of BSE**						
No	Ref	–	> 0.001	Ref	–	0.004
Yes	0.32	0.22, 0.46		0.59	0.42 , 0.85	
**Delay time (days)**	5.57	1.75, 10.55	> 0.001	3.25	1.04, 5.21	0.001
**Type of first symptom**						
Lump	Ref	–	–	Ref	–	–
Discharge and pain	2.09	1.47, 2.98	> 0.001	1.61	1.12, 2.32	0.01
Other^e^	2.76	1.74, 4.31	> 0.001	1.13	0.69, 1.85	0.60
**Type of tumor**						
Ductal	Ref	–	–	Ref	–	–
Lobular	1.33	0.89, 2.00	0.162	1.06	0.69, 1.63	0.77
Other^f^	4.40	2.76, 6.99	0.001	2.11	1.25, 3.57	0.005
**Location**						
Right	Ref	–	0.174	Ref	–	< 0.001
Left	1.23	0.91, 1.68		2.64	1.88 , 3.71	
**Chronic disease**						
No	Ref	–	0.073	Ref	–	0.006
Yes	1.05	0.77, 1.44		1.61	1.14 , 2.26	
**Smoking**						
No	Ref	–	0.156	Ref	–	0.03
Yes	1.36	0.88, 2.09		1.59	1.36 , 1.97	

^a^Based on univariate logistic regression.

^b^According to the multiple logistic regression (adjustment for all the study variables).

^c^Natural menopause.

^d^First and/or second relatives.

^e^Such as itch, rush and bleeding.

^f^Including mucinous, medullary and not identified types of tumor.

In the multivariable analysis, those who had a longer delay in diagnosis (day) were more likely to be diagnosed with end-stage tumor of BC (aOR 3.25, 95% CI 1.04–5.21). Also, the first symptoms reported by the patient (aOR discharge or pain vs. lump 1.61, 95% CI 1.12–2.32) and histological subtype (aOR other vs. ductal 2.11, 95% CI 1.25–3.75) were significantly associated with end-stage of BC. On the other hand, those with tumor in their right breast (aOR right vs. left 2.64, 95% CI 1.88–3.71) and those with a history of chronic disease (aOR 1.61, 95% CI 1.14–2.26) and smokers (aOR no vs. yes 1.59, 95% CI 1.36–1.97) were at a higher risk of being diagnosed with an end-stage tumor (Table 2).

### The association between the study variables and delay in BC diagnosis

Older age at diagnosis was significantly associated with patients delay in diagnosis (aOR 50–60 years vs. < 40 years: 2.04, 95% CI 1.40–3.65) but not doctor delay (aOR 50–60 years vs. < 40 years 1.22, 95% CI 0.80–6.12). Similarly, older age at marriage was only associated with patients delay (aOR > 30 vs. < 20: 2.90, 95% CI 1.32–6.40) (Table 3). Obesity was associated with patients delay in diagnosis (aOR overweight vs. normal 2.35, 95% CI 1.22–4.55). Higher income was inversely associate with patients’ delay in diagnosis (aOR high vs. low-income: 0.48, 95% CI 0.27–0.87), but not doctor delay (aOR high vs. low-income 0.77, 95% CI 0.0.27–2.15) (Table 3). Likewise, knowledge of BSE was inversely associated with patients’ delay (aOR yes vs. no: 0.40, 95% CI 0.25–0.63). Having health insurance was associated with a shorter delay in diagnosis for both patients (aOR yes vs. no 0.50, 95% CI 0.30–0.86) and doctor delay (aOR yes vs. no 0.21, 95% CI 0.10–0.44). Similarly, living in urban areas was inversely associated with both patient’s delay (aOR yes vs. no 0.53, 95% CI 0.34–0.83) and doctor’s delay (aOR yes vs. no 0.41, 95% CI 0.20–0.84). History of benign breast disease was associated with patient delay (aOR 1.67, 95% CI 1.07–2.59) and doctor’s delay (aOR 3.30, 95% CI 1.65–6.62). History of a chronic disease was associated with longer delay for both patients (aOR yes vs. no 2.22, 95% CI 1.42–3.45) and doctors (aOR 3.25, 95% CI 1.76–6.36). First symptoms were associated with both patients (aOR discharge or pain vs. lump 1.88, 95% CI 1.17–3.02) delay or doctor delay (aOR other vs. lump 2.71, 95% CI 1.08–6.80).

**Table 3:** Results of multiple regression analyses on the association between study variables and delay in diagnosis (N = 750) among Iranian women based on rote of delay. 2017–2019.

Variables	Overall Delay			Patient delay			Doctor delay		
	OR	95% CI	P value	OR	95% CI	P value	OR	95% CI	P value
**Age (years)**									
< 40	Ref	–	–	Ref	–	–	Ref	–	–
40–50	1.25	0.80, 1.96	0.33	1.15	0.67, 1.10	0.61	1.03	0.37, 2.83	0.95
50–60	1.97	1.21, 3.20	0.006	2.04	1.40, 3.65	0.02	2.22	0.80, 6.12	0.12
> 60	1.58	0.74, 3.40	0.23	1.90	0.81, 4.47	0.14	1.51	0.26, 8.77	0.64
**Family income**									
Low	Ref	–	–	Ref	–	–	Ref	–	–
Moderate	0.42	0.27, 0.67	< 0.001	0.21	0.11, 0.39	< 0.001	1.37	0.52, 3.60	0.51
High	0.86	0.55, 1.35	0.52	0.48	0.27, 0.87	0.01	0.77	0.27, 2.15	0.62
**Health insurance**									
No	Ref	–	–	Ref	–	–	Ref	–	–
Yes	0.53	0.34, 0.82	0.004	0.50	0.30, 0.86	0.01	0.21	0.10, 0.44	< 0.001
**Place of residence**									
Rural	Ref	–	–	Ref	–	–	Ref	–	–
Urban	0.40	0.28, 0.58	< 0.001	0.53	0.34, 0.83	0.005	0.18	0.09, 0.37	< 0.001
**Marriage status**									
Single (never married)	Ref	–	–	Ref	–	–	Ref	–	–
Ever married	0.62	0.42, 0.92	0.01	0.61	0.37, 0.99	0.04	0.41	0.20, 0.84	0.01
**Marriage age**									
< 20	Ref	–	–	Ref	–	–	Ref	–	–
20–25	1.33	0.74, 2.42	0.34	1.51	0.76, 2.99	0.23	0.84	0.19, 3.63	0.82
25–29	2.53	1.42, 4.50	0.002	3.06	1.51, 6.21	0.002	2.19	0.62, 7.65	0.21
> 30	1.80	0.94, 3.43	0.07	2.90	1.32, 6.40	0.008	0.48	0.12, 1.95	0.31
Not married	1.36	0.85, 2.18	0.20	1.71	0.98, 2.98	0.05	1.56	0.53, 4.60	0.41
**Menopause status**									
Postmenopausal	Ref	–	–	Ref	–	–	Ref	–	–
Premenopausal	0.24	0.17, 0.36	< 0.001	0.28	0.16, 0.46	< 0.001	0.08	0.03, 0.21	< 0.001
**History of breast problem**									
No	Ref	–	–	Ref	–	–	Ref	–	–
Yes	1.88	1.30, 2.73	0.001	1.67	1.07, 2.59	0.02	3.30	1.65, 6.62	0.001
**Aware of BSE**									
No	Ref	–	–	Ref	–	–	Ref	–	–
Yes	0.55	0.37, 0.82	0.003	0.40	0.25, 0.63	< 0.001	1.61	0.55, 4.72	0.37
**Type of first symptom**									
Lump	Ref	–	–	Ref	–	–	Ref	–	–
Discharge and pain	1.92	1.29, 2.87	0.001	1.88	1.17, 3.02	0.008	2.07	0.97, 4.40	0.05
Other	1.81	1.06, 3.10	0.03	1.51	0.76, 2.98	0.23	2.71	1.08, 6.80	0.03
**Type of tumor**									
Ductal	Ref	–	–	Ref	–	–	Ref	–	–
Lobular	1.12	0.70, 1.78	0.64	1.42	0.78, 2.58	0.24	0.43	0.18, 1.03	0.06
Other	2.05	1.17, 3.60	0.01	1.96	0.84, 4.58	0.11	2.08	0.96, 4.51	0.06
**BMI (kg/m**^**2**^**)**									
Normal (18.50–24.99)	Ref	–	–	Ref	–	–	Ref	–	–
Overweight (24.99–29.99)	1.74	1.01, 2.10	0.4	2.35	1.22, 4.55	0.01	0.98	0.29, 3.34	0.98
Obese (≥ 30.00)	1.11	0.61, 2.02	0.74	1.85	0.89, 3.81	0.09	0.31	0.08, 1.14	0.08
**Chronic disease**									
No	Ref	–	–	Ref	–	–	Ref	–	–
Yes	2.47	1.71, 3.56	< 0.001	2.22	1.42, 3.45	< 0.001	3.35	1.76, 6.36	< 0.001

## Discussion

The results of the present study indicate that living in rural areas, being single, post-menopausal, family history of BC, history of breast benign disease, lack of knowledge about BSE, delay at diagnosis, having discharge and pain as the initial signs of BC, tumor located in left breast, history of chronic diseases and smoking are significantly associated with an increased risk of end-stage diagnosis of BC. Moreover, overall, age at diagnosis, low family income, lack of health insurance, rural residential, being single, post-menopausal, lack of knowledge about BSE, having discharge and pain as the initial signs of BC, high BMI and history of chronic disease were significantly associated with delay at diagnosis.

Previous studies have shown significant differences and disparities in the diagnosis and treatment of BC between women from urban and rural areas worldwide, indicating that living in rural areas is associated with late-stage diagnosis of BC^23,24^. In our study, living in rural area was associated with both delay at diagnosis and higher risk of end-stage at diagnosis, which may be attributed to limited access of women from rural areas to screening and diagnostic services^25^. Our finding that living in rural areas is associated with delay in BC diagnosis is in line with previous studies^18,26^. It is suggested that distance from medical services is also associated with doctor’s delay^17^, and that rural areas need to be empowered in terms of training, education and improvement in access to health services with a particular emphasis on BC^27^. Moreover, women in rural areas are more likely to turn to traditional and alternative therapies in the end stages of the disease, due to long distances to medical facilities and lack of access to breast cancer diagnosis and treatment services^28^.

It has been reported that low health insurance coverage is one of the major contributors to system delays and consequently detection at end stages of the disease^18,29^. A study found that uninsured women reported lower rates of mammography screening than other women. Also, women with only public insurance, were less likely than women with medicare and private to undergo mammography screening^30^. Furthermore, lower-income and unemployed women may not prioritize access to health services, including breast cancer screening. As a result, uninsured women are less likely to have access to breast cancer screening than insured women. In some countries, health insurance may provide women with use of health care services, regardless of their financial status^28,31^.

The knowledge of BSE or performing BSE was associated with decreased odds of both end-stage BC diagnosis and delay at diagnosis in our study. The literature is inconclusive regarding the role of BSE on early diagnosis of BC, as some studies found no association between breast self-examination and delay in BC diagnosis^32^, while it was associated with a reduced risk of patient delay in some others^16^. The results of various review studies suggest that BSE can be a primary BC screening method because women will be aware of their physical changes earlier. However, these studies revealed poor awareness of women regarding this method, and establishment of training interventions has been emphasized in these studies^13,33^.

Our findings suggest that compared to single women, married women were at a lower risk of delay at diagnosis. Various studies have highlighted the potential role of marriage in providing psychological benefits, economic resources, and social support in the early detection of cancer as well as the treatment and survival of cancer patients^12,34,35^.

In our study, post-menopausal women were at a higher risk of end-stage diagnosis. A study in India found that end-stage at diagnosis was common in both pre- and post-menopausal women, with a higher number of post-menopausal women with stage 4 BC^14^. In accordance with the literature, our study suggest that older age is an important factor associated with longer patient-related delay^16,26^. A study in India reported that women aged less than 40 years were less likely to comply with medical advice^32^, and the highest delay (49%) was in women aged 40–50 years. These findings were in compliance with reports from Iran^36–38^, but not other countries^39,40^.

In our study, previous history of benign breast conditions was significantly associated with long diagnosis delay. BC patients often consider breast problems as benign, which is an important cause of patient delay^41^. Also, in a report, breast symptoms were significantly correlated with physician delay^17^. The evidence suggests that all women should be vigilant of any change in their bodies and consider undergoing validated diagnostic tests, annual specialized examinations, and screening programs. Besides, our findings are in line with previous reports that people with invasive lobular carcinoma (ILC) experience worse prognosis compared with those with invasive ductal carcinoma (IDC)^15,42^, and that ILC is less common but more difficult to diagnose with mammography, and is usually diagnosed at an end stage^43^. Regarding signs of BC, our results showed that discharge and pain were associated with end-stage diagnosis compared to those with a lump. It was reported that a considerable proportion of patients ignored the clinical significance of the early symptoms and attributed these symptoms to other nonspecific conditions, and that breast mass was the first symptom associated with shorter detection and delay in diagnosis^44,45^.

We found an increased risk of end-stage at diagnosis among smokers and those with chronic conditions. A study in Iran found that women with chronic diseases were more likely to be diagnosed at a late-stage BC^46^, which was in compliance with our results. Patients with chronic conditions are likely to attribute BC symptoms to their comorbidities, thus, miss the chances for early detection. However, the evidence is scarce and further research is required. It is suggested that smokers are less likely to participate in BC screening^47^, and thus are at a decreased chance of early diagnosis^48^.

Interestingly, in our study overweight women were twice more likely to have delayed diagnosis compared to women with normal weight. Obesity was reported to be strongly associated with delay in BC diagnosis compared with other health related variables in studies conducted in Germany^39^ and the USA^49^. It is suggested that larger breasts in overweight/obese women can hinder tumor detection and result in delay at diagnosis^49^. In addition, obesity may be associated with an end-stage at diagnosis because of an underlying endocrinologic abnormality related to tumor progression, including levels of sex hormone‐binding globulin and estradiol^49^. Obesity causes an increased production of the estrogen known as estrone via the aromatization of androstenedione in peripheral adipose tissue^50^. The age‐adjusted prevalence of overweight or obesity (BMI ≥ 25) among Iranian women is 57.0%^51^. Thus, considering the high and increasing prevalence of obesity in women in the Iran, our findings are of particular concern.

The findings of various studies have shown that delayed diagnosis is associated with end stages of the disease that may occur by physicians, the treatment system or the patient. Unger-Saldana and colleagues showed that 41% of women who were finally diagnosed with cancer, were detected as benign at their first medical consultation, which can lead to a lack of patient trust in the healthcare system. As a result, improved quality of primary care, prompt hospital referral system, improved patient information and physician education and training are required to promote early diagnosis of BC^52,53^.

Diagnosis of late-stage BC is a major challenge in the Iranian community. Thus, multi-sectorial approach and appropriate strategies aimed at early detection and effective management of the disease is important to reduce the burden of BC^14^.

A review of 53 studies (24 were carried out in developing countries and 29 in developed countries) suggested that studies conducted in developing countries were more focused patient delay and its determinants, while, studies conducted in developed countries were mainly focused on system delay during treatment and guidance of breast cancer patients in the health care system^54^. The greater focus on patient delay in developing countries may stem from the hypothesis that patients in these countries are not well aware of the risk factors for breast cancer. The factors affecting patient and system delays depend on the patient's social and cultural environment and differences in health care systems and the patients personal decisions^54^.

The ability to collect information on socio-economic status, knowledge and practice of breast self-examination and some clinical factors in a relatively large sample of newly diagnosed patients in two major centers in the country were strengths of this study. Recruiting participants visited the biggest referral centers in Iran makes the results generalizable to the population. Also, recruiting new cases of BC minimize the chance of recall bias in this study. However, the possibility of error in self-reported information can not be rejected as some women may not have reported correctly the reason of delay or the type of symptoms or the time that the first symptom was noticed. Previous studies suggested that the information regarding delay time and symptoms of breast cancer seem to be fairly precise^22,39^. Another limitation was the lack of information on the status of oestrogen (ER) and progesterone (PR) hormone receptor and human epidermal growth factor receptor 2 (HER2).

In conclusion, our study suggests that factors including history of BBD, knowledge of BSE, and comorbidity were associated with both delay in diagnosis and end stage of disease. Our findings have important implications, urging physicians and health care providers to take extra caution when a woman with BBD refer to the breast clinics. Also, the implementation of educational programs is likely to increase women's awareness of their anatomy, and might help earlier diagnosis.

## Methods

### Ethics statement

The protocol of this study was reviewed and approved by the Ethics Committee affiliated with Shiraz University of Medical Sciences (approval code: 97-01-69-17629). The study subjects were informed about the study process and confidentiality of data and provided oral informed consent.

### Settings

This study was conducted and reported in accordance with the STROBE (STrengthening the Reporting of OBservational studies in Epidemiology) statement^55^. The present study was a hospital-based cross-sectional study, which included a total of 725 female BC patients newly diagnosed (incident cases) at two referral centers in Iran; Imam Reza Hospital in Namazi Hospital in Shiraz, Southern Iran (n = 460) and Kermanshah city located in the West of Iran (n = 265) from June 2017 to December 2019.

### Sampling and inclusion criteria

Power analysis suggested that with such a sample size a significant level at 5% and 90% power, 50% difference in the risk of late stage diagnosis was detectable for those being aware of BSE.

Only newly diagnosed (< 6 months)^46^ patients who had pathology reports were invited. Patients with history of cancer or relapsed disease were excluded from the study. Also, participants with mental disorders or with impaired hearing were excluded.

### Data collection

A validated questionnaire^22^ was used during a face-to-face interview by a trained female nurse to obtain information on socio-demographic factors including age, education (primary and lower, middle school, high school, college), age at first marriage (year), marital status (single, married), occupation (employed, housewife), menopausal status (pre-, post-menopausal), residency (rural, urban), health insurance (yes, no), daily exercise (< 10 min, 10–20 min, > 20 min), body mass index (BMI) (kg/m^2^) (underweight, normal, overweight, obese), smoking (yes, no), X-ray history (yes, no), chronic disease (yes, no), delay time (day), family history of BC (yes, no), age at first pregnancy (less than 20 years, between 20 and 30 years, more than 30 years and never married or nulliparous), history of BBD (yes, no), and the status of knowledge and regular practice of BSE (yes, no) and clinical factors including nodal status which was collected from the records (N−, N + , other), type of first symptom (lump, discharge and pain, other), location of tumor (right, left), tumor type (ductal, lobular/ medullary, others), self-reported date and type of initial sign and symptom of BC noticed by the patients, and also date of first symptom recognition and recall the month and year of their first medical consultation due to BC; this date was used as a reference to questions about whether or not she perceived symptoms, the time symptoms were present before first consultation and socioeconomic factors at the moment of first medical consultation. Moreover, the main reason for the delay in diagnosis was also reported by the participants. Clinical data were collected by reviewing the patients’ medical records by an experienced medical coder. Clinical data including stage of disease, tumor size and lymph node status also were extracted from patients’ medical records.

### Outcomes

Diagnosis delay (day) was the primary outcome; defined as the interval between the date that patient noticed the first symptom until the date of histological diagnosis. The reasons for delay, reported by the patients, were divided into two categories: patient interval, which was defined as the time from experiencing the symptoms to the first medical consultation; and provider interval, which was defined as the time from the first presentation (first medical consultation) to the beginning of cancer treatment. Our secondary outcome was the stage at diagnosis, which was defined according to the tumor, node, and metastasis classification system (TNM staging system)^56^. Patients were classified as having either early stage disease (stage I/II), or end-stage (locally advanced disease (stage III) or metastatic disease (stage IV)) at the time of diagnosis^53^.

### Statistical analysis

To impose the clinical importance of diagnostic delay in bivariate analysis, the delay time was categorized to less or equal (no diagnostic delay) and longer (diagnostic delay) than 3 months^46^. For bivariate analysis, the unadjusted associations of all independent variables with the stage of cancer were measured by the chi-square test. Strength of the association was measured using odds ratio (OR) and 95% confidence intervals. Adjusting for all the mentioned above variables, multivariable logistic regression was used to measure the adjusted associations between the study variables and cancer stage. Also, we used linear regression to find associations between study variables and delay in diagnosis (day). Statistical analysis was conducted assuming two-sided 5% level of significance. STATA (STATA Corp. version 14.2) was used for analysis the data.

## Data Availability

All data that was obtained and analyzed during this study are included in this article.

## Abbreviations

BC: Breast cancer
BBD: Benign breast disease
BSE: Breast self-examination
IDC: Invasive ductal carcinoma
ILC: Invasive lobular carcinoma
TNM: Tumor, nodes, and metastases
BMI: Body mass index
OCP: Oral contraceptive pills
SD: Standard deviation
aOR: Adjusted odds ratio
95% CI: 95% Confidence interval
